# Mass Spectrometry Identification of Biomarkers in Extracellular Vesicles From *Plasmodium vivax* Liver Hypnozoite Infections

**DOI:** 10.1016/j.mcpro.2022.100406

**Published:** 2022-08-24

**Authors:** Melisa Gualdrón-López, Miriam Díaz-Varela, Gigliola Zanghi, Iris Aparici-Herraiz, Ryan W.J. Steel, Carola Schäfer, Pol Cuscó, Vorada Chuenchob, Niwat Kangwangransan, Zachary P. Billman, Tayla M. Olsen, Juan R. González, Wanlapa Roobsoong, Jetsumon Sattabongkot, Sean C. Murphy, Sebastian A. Mikolajczak, Eva Borràs, Eduard Sabidó, Carmen Fernandez-Becerra, Erika L. Flannery, Stefan H.I. Kappe, Hernando A. del Portillo

**Affiliations:** 1 ISGlobal, Barcelona Institute for Global Health, Barcelona, Spain; 2 IGTP, Institute for Health Sciences Trias I Pujol, Barcelona, Spain; 3 Center for Global Infectious Disease Research, Seattle Children’s Research Institute, Seattle, Washington, USA; 4 Department of Pathobiology, Faculty of Science, Mahidol University, Bangkok, Thailand; 5 Department of Laboratory Medicine and Pathology, and Department of Microbiology, University of Washington, Seattle, Washington, USA; 6 MVRU, Mahidol Vivax Research Unit, Mahidol University, Bangkok, Thailand; 7 Centre for Genomic Regulation (CRG), The Barcelona Institute of Science and Technology, Barcelona, Spain; 8 Universitat Pompeu Fabra (UPF), Barcelona, Spain; 9 Department of Pediatrics, University of Washington, Seattle, Washington, USA; 10 ICREA, Catalan Institution for Research and Advanced Studies, Barcelona, Spain

**Keywords:** *Plasmodium vivax*, humanized mouse model, hypnozoites, extracellular vesicles, proteomics, biomarkers, schizonticidal experimental drug MMV048, AEpS, abundance estimated per sample, D8, Day 8 postinfection, D21, Day 21 postinfection, DMSO, Dimethyl sulfoxide, EVs, Extracellular vesicles, FRG KO (FRG huHEP), C57Bl/6 mice with a triple mutation (FAH−/−Rag2−/−IL2Rγnull) and repopulated with primary human hepatocytes, ID, identifier, IFA, Immunofluorescence assay, IV, intravenous infection, MB, mosquito bite, PI4K, phosphatidylinositol kinase, PIK3C2B, phosphatidylinositol-4-phosphate 3-kinase catalytic subunit type 2 beta, PR, protein ratio, SEC, size-exclusion chromatography, TBS, Tris-buffered saline, TQ, tafenoquine

## Abstract

Latent liver stages termed hypnozoites cause relapsing *Plasmodium vivax* malaria infection and represent a major obstacle in the goal of malaria elimination. Hypnozoites are clinically undetectable, and presently, there are no biomarkers of this persistent parasite reservoir in the human liver. Here, we have identified parasite and human proteins associated with extracellular vesicles (EVs) secreted from *in vivo* infections exclusively containing hypnozoites. We used *P. vivax*-infected human liver-chimeric (huHEP) FRG KO mice treated with the schizonticidal experimental drug MMV048 as hypnozoite infection model. Immunofluorescence-based quantification of *P. vivax* liver forms showed that MMV048 removed schizonts from chimeric mice livers. Proteomic analysis of EVs derived from FRG huHEP mice showed that human EV cargo from infected FRG huHEP mice contain inflammation markers associated with active schizont replication and identified 66 *P. vivax* proteins. To identify hypnozoite-specific proteins associated with EVs, we mined the proteome data from MMV048-treated mice and performed an analysis involving intragroup and intergroup comparisons across all experimental conditions followed by a peptide compatibility analysis with predicted spectra to warrant robust identification. Only one protein fulfilled this stringent top-down selection, a putative filamin domain-containing protein. This study sets the stage to unveil biological features of human liver infections and identify biomarkers of hypnozoite infection associated with EVs.

## Highlights

• Use of *in vivo Plasmodium vivax* model to study latent liver infections.
• Use of the MMV048 drug to selectively kill replicating liver parasites.
• Proteome of human hepatocyte-specific EVs present in a complex biofluid.
• EVs from this model contain parasite proteins, one exclusively associated with hypnozoites.

## Graphical Abstract

**Figure:**
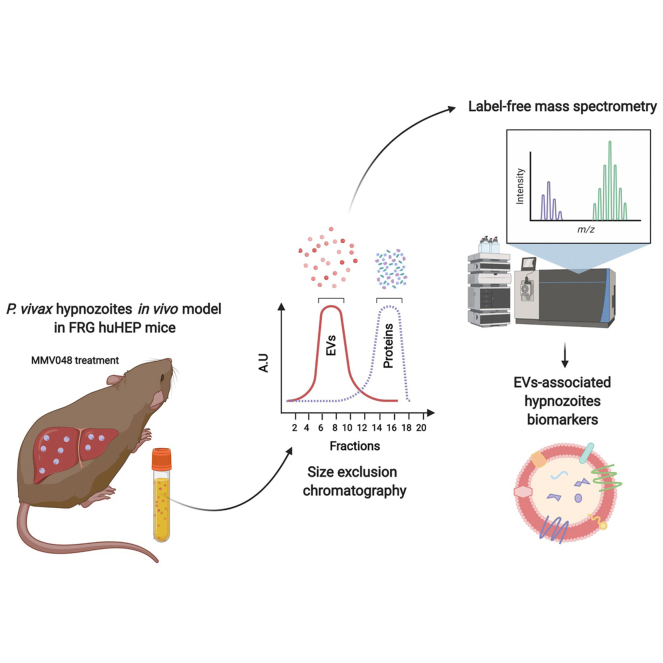

## In Brief

Human malaria caused by *Plasmodium vivax* represents a major barrier to achieve the goal of malaria elimination as this species evolved a dormant liver form called a hypnozoite that can remain latent after the initial infection. Current diagnostic tools are unable to detect asymptomatic patients harboring hypnozoites. We have used a MS proteomics approach to discover biomarkers of latent liver infections associated with circulating extracellular vesicles in an *in vivo* model of *P. vivax* liver hypnozoite infections.

*Plasmodium vivax* is the most widely distributed malaria parasite responsible for the majority of malaria cases outside sub-Saharan Africa with 2.5 billion people at risk (1). This parasite causes close to seven million malaria cases yearly distributed in Central and Southeast Asia, Americas, and Eastern parts of Africa. Although *P. vivax* causes less mortality than *Plasmodium falciparum*, severe disease and complicated malaria are also attributable exclusively to this species (2, 3). The biology of *P. vivax* is complex and differs in several aspects from *P. falciparum* making it difficult to eliminate *P. vivax* infections with similar control and treatment strategies. After transmission of infectious sporozoite stages by mosquito bite (MB), *P. vivax* is characterized by the formation of latent liver-stage forms derived from some sporozoites called hypnozoites (4). These nonreplicating forms can persist in the human liver for months to years and then reactivate to undergo schizogony and produce infectious merozoite forms that initiate recurrent and clinically manifest blood infections called relapses. Numerous studies indicate that relapses are responsible for up to 90% of all *P. vivax* malaria cases in several endemic countries (5, 6, 7). The presence of hypnozoites in the human population thus constitutes a major challenge to the World Health Organization malaria eradication goal. Individuals harboring silent hypnozoites are not only suffering recurrent blood-stage infections but they are also the source for continued parasite transmission.

In recent years *P. vivax* hypnozoite biology has started to be unveiled, owing to the development of new technologies. The construction of a humanized liver chimeric FRG huHEP mouse *in vivo* model for liver stages (8, 9), together with optimized primary human hepatocytes *in vitro* culture systems (10), including micropatterned cocultures (11) and more recently liver spheroids (12), have allowed to uncover unknown aspects of hypnozoites. Thus, transcriptional analysis of *in vitro* cultured *P. vivax* (13) and simian *Plasmodium cynomolgi* hypnozoites (14, 15, 16) has revealed gene expression patterns indicating that mature hypnozoites have a reduced transcriptional activity. Yet, dormant hypnozoites express genes involved in energy metabolism, transcriptional, and translational control, protein export, quiescence, and maintenance of genome integrity. Moreover, *P. vivax* infection in FRG huHEP mice have shown that hypnozoites perform active cellular processes such as endoplasmic reticulum biogenesis as well as apicoplast and mitochondrial replication (8). More recently, the creation of a dual reporter *P. cynomolgi* cell line allowed the observation of individual hypnozoites transitioning to replicating schizonts, a major breakthrough in hypnozoite biology and a unique platform for the screening of putative antirelapse drugs (15, 16).

Extracellular vesicles (EVs) are double membrane nanovesicles secreted by all cell types that are involved in intercellular communication (17). These vesicles originated from different cell compartments, including multivesicular bodies (exosomes) and plasma membrane (microvesicles, small vesicles, oncosomes, and apoptotic bodies), and shows high heterogeneity in size and molecular composition (18). The molecular content of EVs (protein, lipids, nucleic acids, and metabolites) reflect the physiological status of the cell of origin (19). This feature, together with the fact that EVs are present in all biological fluids so far studied, has prompted its exploration as biomarkers in liquid biopsies of a wide range of pathologies, including cancer (20), neurological disorders (21), diseases affecting lungs (22), kidney (23), liver (24), as well as infectious diseases (25).

Numerous studies have demonstrated that malaria-infected cells secrete EVs that contain parasite proteins and are involved in host–parasite interactions (26, 27, 28). These include cell–cell communication (29, 30), modulation of immune responses (31, 32), alterations of vascular endothelium (33, 34), cerebral malaria pathogenesis (35), and induction of adhesion molecules in spleen fibroblast (36), among others. While most studies have been focused on vesicles derived from blood-stage parasites, it remains to be determined if EVs derived from hepatocytes infected with *Plasmodium* liver stages have any function in intercellular communication and can identify biomarkers of latent *P. vivax* liver infection. We have previously demonstrated that plasma-derived EVs isolated from the liver-stage model of *P. vivax*-infected FRG huHEP mice contain parasite proteins (37) indicating the potential of this model for discovering hypnozoite biomarkers. However, a limitation of our previous study was that livers of infected FRG huHEP mice contained both replicating schizonts together with hypnozoites, precluding us from distinguishing EVs exclusively derived from hypnozoite-infected hepatocytes. Here, we have employed an experimental approach exploiting the schizonticidal properties of the experimental drug MMV048 to generate *in vivo* infections of *P. vivax* hypnozoites to explore the protein content of EVs derived from hypnozoite-infected hepatocytes.

## Experimental Procedures

### Experimental Design and Statistical Rationale

**Fig. 1:**
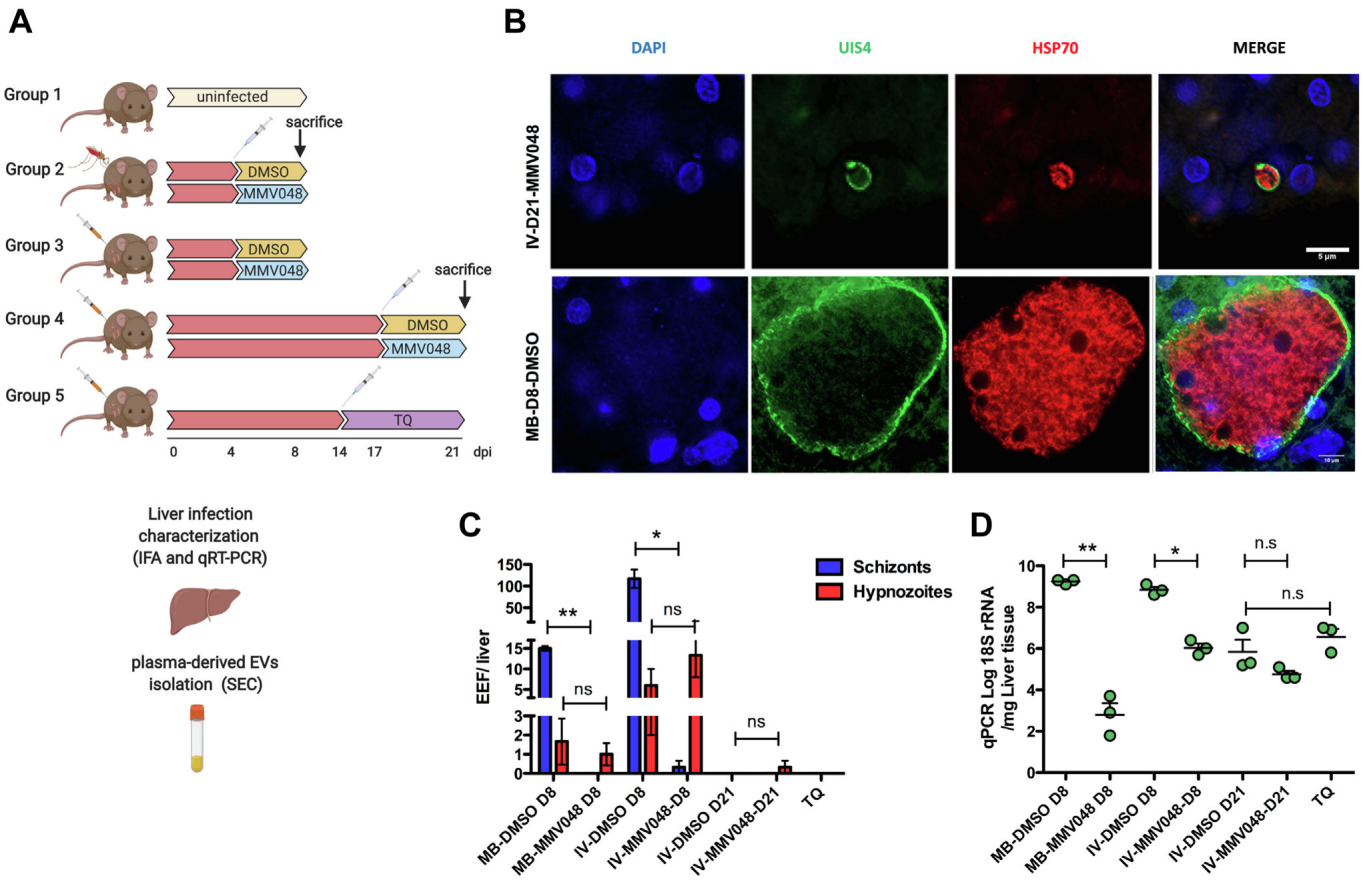
**Characterization of EVs from plasma of *P. vivax*-infected FRGhuHEPmice.***A*, schematic representation of experimental groups of *P. vivax*-infected FRG huHEP mice treated with MMV048. Three mice were used per treatment in each group. Group 1 corresponds to uninfected FRG huHEP mice. Group 2 corresponds to mice infected by mosquito bite and treated at day 4 postinfection with MMV048 intravenously or DMSO as control. Animals from this group were euthanized at 8 dpi. Group 3 and 4 correspond to mice intravenously infected with *P. vivax* sporozoites and treated at 4 (group 3) and 17 days (group 4) postinfection with MMV048 intravenously or DMSO as control. Mice from these two groups were euthanized at 8 and 21 days, respectively. Group 5 corresponds to mice intravenously infected with *P. vivax* sporozoites, treated at 14dpi with tafenoquine intravenously and euthanized at 21 dpi. *B*, characterization of *P. vivax* liver infection in FRG huHEP mice treated with MMV048. Representative image of IFA analysis showing an hypnozoite form in the liver of intravenously infected (IV) MMV048-treated mice from group 4 (*top*) and a schizont form in the mosquito bite infected (MB) DMSO-treated control mice from group 2 (*bottom*). Scale bar in *top* = 5 μm and in *bottom* = 10 μm. *C*, quantification of liver stages by IFA. *D*, quantification of parasite load by RT-qPCR (log10 plasmodium 18S rRNA per μg liver RNA). Data represent mean and standard error of measurements performed in individual mice. Statistical significance was assessed by paired *t* test ∗*p* < 0.05, ∗∗*p* < 0.01. *A* has been created with BioRender.com. EVs, extracellular vesicles; IFA, immunofluorescence assay.

To identify *P. vivax* hypnozoites biomarkers associated with EVs, we employed an *in vivo* liver infection model using *P. vivax*-infected FRG huHEP mice treated with the schizonticidal drug MMV048. We isolated plasma-derived EVs from three biological replicate of each experimental group as shown in Figure 1*A* and detailed in the section “P. vivax Infection of FRG huHEP Mice”.

### *P. vivax* Infection of FRG huHEP Mice

All animal procedures were conducted in accordance with and approved by the Center for Infectious Disease Research Institutional Animal Care and Use Committee (IACUC). The Center for Infectious Disease Research IACUC adheres to the NIH Office of Laboratory Animal Welfare standards (OLAW welfare assurance #A3640-01). Female FRG KO mice engrafted with human hepatocytes (FRG KO huHEP) were purchased from Yecuris Corporation. *P. vivax* infections of FRG huHEP were performed as previously described (8). Briefly, mice were divided into five experimental groups. Group 1 (three mice) was not infected. Group 2 (six mice) was inoculated with *P. vivax* sporozoites by the bite of 20 mosquitos and euthanized after 8 days postinfection (dpi). Group 3 (six mice) was infected by intravenous injection of *P. vivax* sporozoites (0.8 million) and euthanized after 8dpi. Group 4 (six mice) was infected by intravenous injection of *P. vivax* sporozoites (1 million sporozoites) and euthanized after 21dpi. Group 5 (three mice) was infected by intravenous injection (1 million sporozoites) and treated with tafenoquine (TQ) (10 mg/kg) at 14 dpi and euthanized at 21 dpi. MMV048 (Novartis) mice treatment (30 mg/kg) was performed as follows: three mice from groups 2 and 3 received intravenous injections of the drug at 4 dpi; three mice from group 4 received intravenous injections of the drug at 17 dpi. Three dimethyl sulfoxide (DMSO)-treated animals were used as controls in groups 2, 3, and 4. After euthanasia, livers were extracted for characterization by immunofluorescence assay (IFA) and RT-qPCR, and whole blood was extracted by cardiac venipuncture for plasma collection as previously described (38).

### Immunofluorescence Analysis

IFA analysis of FRG huHEP mice liver sections was performed as described (9, 38). Briefly, liver tissue was fixed in 4% paraformaldehyde in tris-buffered saline (TBS) for 16 h. FRG huHEP-fixed livers were sectioned in 50 μm thick sections in a vibratome. Tissue sections were then permeabilized in 0.25% Triton X-100 and 3% H_2_O_2_ for 30 min followed by a blocking step in 5% skim milk in TBS for 1 h at RT. Mice livers double staining was performed using rabbit anti-*P. vivax* HSP70 primary antibodies and a mouse monoclonal anti-*P. vivax* UIS4 antibody. Fluorescent staining was achieved by incubation with Alexa Fluor-conjugated secondary antibodies (Thermo Fisher) specific to rabbit (Alexa Fluor 594) and mouse (Alexa Fluor 488) IgG for 2 h at RT. After one wash in TBS, nuclei were stained with 4,6-diamidino-2-phenylindole for 10 min at RT and samples mounted with ProLong anti-fade Mountant (Thermo Fisher). Images were acquired using Olympus 1x70 DeltaVision deconvolution microscopy.

### 18S qRT-PCR Analysis of Parasite Load

Liver parasite load from infected FRG huHEP mice was quantified as previously described (9).

### Isolation of EVs

Plasma-derived EVs from uninfected and *P. vivax*-infected MMV048 treated or untreated mice were thawed on ice and centrifuged at 2000*g* for 10 min. EVs were purified by size exclusion chromatography (SEC) using commercial Sepharose (q-EV iZON) following manufacturer instructions. SEC fractions were characterized by bead-based flow cytometry (39) for the presence of CD9 (Abcam ab92726) and CD5L (human plasma EV marker) (Abcam ab45408). CD9^+^ CD5L^+^SEC fractions were further tested for CD63, CD81, and HLA-I by bead-based flow cytometry using the following antibodies: anti-human CD63, (Immunostep 63PU-01MG), hybridoma anti-human CD81 (clone 5A6), and anti-human HLA-ABC, (Invitrogen 14-9983-82). In addition, EVs-enriched SEC fractions were analyzed by nanoparticle tracking analysis and cryo-electron microscopy. Protein concentration was determined by BCA (Thermo Scientific).

### Liquid Chromatography Tandem Mass Spectrometry

Hundred microliter of highest CD9 and CD5L abundance SEC fractions from *P. vivax*-infected FRG huHEP mice plasma–derived EVs were processed for peptide digestion using commercial kit (PreOmics) according to the manufacturers’ protocol adapted for samples containing <20 μg of protein. For comparison, we normalized the amount of sample to be analyzed by LC-MS/MS relative to the amount of FRG huHEP mice plasma processed for EVs isolation. Between 0.8 and 2 μg of each sample were analyzed using an LTQ-Orbitrap Fusion Lumos mass spectrometer (Thermo Fisher Scientific) coupled to an EASY-nLC 1200 (Thermo Fisher Scientific (Proxeon)) at the Proteomics Unit of the Center for Genomic Regulation (Spain). Peptides were loaded directly onto the analytical column and were separated by reversed-phase chromatography using a 50-cm column with an inner diameter of 75 μm, packed with 2 μm C18 particles spectrometer (Thermo Scientific). Chromatographic gradients started at 95% buffer A and 5% buffer B with a flow rate of 300 nl/min for 5 min and gradually increased to 25% buffer B and 75% A in 79 min and then to 40% buffer B and 60% A in 11 min. After each analysis, the column was washed for 10 min with 10% buffer A and 90% buffer B, buffer A: 0.1% formic acid in water and buffer B: 0.1% formic acid in 80% acetonitrile. The mass spectrometer was operated in positive ionization mode with nanospray voltage set at 2.4 kV and source temperature at 275 °C. The acquisition was performed in data-dependent acquisition mode, and full MS scans with one micro scans at resolution of 120,000 were acquired over a mass range of m/z 350 to 1500 with detection in the Orbitrap mass analyzer. In each cycle of data-dependent acquisition analysis, following each full MS scan, the most intense ions above a threshold ion count of 10,000 were selected for fragmentation. The number of selected precursor ions for fragmentation was determined by the “Top Speed” acquisition algorithm and a dynamic exclusion of 60 s. Fragment ion spectra were produced *via* high-energy collision dissociation fragmentation at a normalized collision energy of 28%, and they were acquired in the ion trap mass analyzer. AGC was set to 10,000, and an isolation window of 1.6 m/z and a maximum injection time of 200 ms were used. Digested bovine serum albumin (New England Biolabs cat # P8108S) was analyzed between each sample to avoid sample carryover and to assure stability of the instrument, and QCloud was used to control instrument longitudinal performance during the project (40). All data were acquired in Xcalibur software, version 3.

### Mass Spectrometry Data Analysis

Acquired spectra were analyzed using the Proteome Discoverer software suite v2.3 (Thermo Fisher Scientific) and the Mascot search engine (v2.6, Matrix Science). The data were searched against a customized database including *P. vivax* (all strains: 52,920 entries) and Swiss-Prot human (20,581 entries) and mouse (17,171 entries) databases (April 2019) plus a list of common contaminants and all the corresponding decoy entries. For peptide identification, a precursor ion mass tolerance of 7 ppm was used for MS1 level, trypsin was chosen as enzyme, and up to three missed cleavages were allowed. The fragment ion mass tolerance was set to 0.5 Da for MS2 spectra. Oxidation of methionine and N-terminal protein acetylation were used as variable modifications, whereas carbamidomethylation on cysteines was set as a fixed modification. A minimum peptide length of 7 was set. FDR in peptide identification was set to a maximum of 1% using a decoy database strategy. Peptide quantification data were retrieved from the “Precursor ion area detector” node from Proteome Discoverer (v2.3) using 2 ppm mass tolerance for the peptide extracted ion current. Protein relative quantification was performed using abundance estimated per sample (AEpS) which is calculated as the average areas of the top three most abundant peptides per protein. Protein ratio (PR) were calculated using the statistical model embedded into ProteomeDiscoverer v2.3 that accounts for all peptide ratios for each of the conditions and provides an adjusted *p* value and an estimate of relative protein quantification measure. Proteins were initially classified according to species *H sapiens*, *M musculus*, and *P. vivax*. *H. sapiens* and *M. musculus* proteins identified with two unique peptides or more were retained. *P. vivax* proteins identified with one unique peptide or more were retained. Parasite proteins were classified according to strains. *P. vivax* proteins identified in the group of uninfected mice were excluded as false positive. Uniprot accession numbers of *P. vivax* proteins were used to retrieve protein sequences and identify their respective ID in Sal I and PVP01 genome through Blast analysis in PlasmoDB (41).

### *P. vivax* Hypnozoite Biomarker Discovery

To identify hypnozoite biomarkers in plasma-derived EVs from *P. vivax*-infected FRG huHEP mice, we first selected all *P. vivax* proteins, irrelevant of the strain and the EV sample. Next, we compared intragroup PR of the three following experimental groups: Group 2: MB-D8-MMV048 *versus* MB-DMSO; group 3: IV-D8-MMV048 *versus* IV-D8-DMSO; and group 4: IV-D21-MMV048 *versus* IV-D21-DMSO. We further excluded proteins present in group 5: IV-TQ-D21 as false-positives. Proteins with a PR >1 and statistical significance (*p* value < 0.01) were considered. In a second attempt to identify other possible candidates overlooked in the previous analysis, we explored AEpS data and down selected proteins that fulfill the previous criteria disregarding statistical significance. Peptide spectra of the selected candidate proteins were inspected manually using Skyline software for their compatibility with the theoretical MS/MS peak intensities predicted by MS2PIP. Finally, we selected only those proteins found exclusively in MMV048-treated animals in an intergroup comparison. Additionally, structural features (presence of transmembrane domains and signal peptides) of candidate proteins were retrieved from PlasmoDB.

### Human Proteome of Plasma-Derived EVs From *P. vivax*-Infected FRG huHEP Mice

We performed a statistical analysis of the human proteome using AEpS data from each experimental group. Briefly, human proteins identified with ≥1 unique peptide and present in more than three mice were accepted for this analysis. Protein levels were log-2 transformed to guarantee data normality. Missing data given the limit of detection were imputed by generating random samples from left truncated lognormal distribution using the R package called truncdist (42). Principal components analysis was used as a quality control of replicates. Linear models were used to assess association between proteins of relevant comparisons. The obtained *p* values were corrected for multiple comparisons using false discovery rate approach in order to avoid false positive results. Comparisons include EVs protein content of *P. vivax*-infected DMSO-treated mice from all groups with content of uninfected mice in order to identify proteins associated with liver infection. In order to identify potential human biomarkers of hypnozoite infections, we performed an intragroup comparison of human proteins from MMV048-treated mice with their respective DMSO-treated control mice in groups 2 and 3. In addition, we also compared DMSO-treated mice from experimental groups 2 and 3 to identify proteins associated with MB or to intravenous infection (IV). A similar comparison was done among human proteins identified in EVs from infected DMSO-treated mice from group 3 and 4 in order to associate proteins to early (8 dpi) and late (21 dpi) infections. Finally, we compared human proteins from EVs from DMSO-treated mice of group 4 with mice treated with TQ in group 5 to identify proteins associated with EVs in radical cure treatment with this drug. All plots have been made with R version 4.1.2 (2021–11–01). Heatmaps were generated using package pheatmap 1.0.12 and volcano plots using the EnhancedVolcano 1.12.0 package.

## Results

### Identification of *P. vivax* Hypnozoites Biomarkers Associated to Plasma-Derived EVs in the FRG huHEP Mice

Five different groups of FRG huHEP mice were used in these studies: uninfected control animals (group 1), mice infected with *P. vivax* sporozoites by mosquitos bite (group 2), intravenous injection and further treated with MMV048 (groups 3, 4), or with the radical cure drug tafenoquine (group 5) (Fig. 1*A*). Considering the previously established infection kinetics of *P. vivax* in the FRG huHEP mice model (8), mature hypnozoites are detected at 8 dpi. In addition, hypnozoites can reactivate and generate a second wave of schizonts at 21 dpi. Taking these observations into account, we performed endpoint analysis at 8 dpi for both, group 2 and 3. In addition, we included an analysis at 21 dpi in mice infected by intravenous injection (Group 4) reasoning that treatment with MMV048 from day 17 to 21 dpi would remove the second generation of schizonts, giving an additional window of time for resistant and growing hypnozoites to secrete EVs in circulation. Liver sections were evaluated by IFA for the presence of UIS4 and HSP70 (8, 9). Results clearly showed small liver stage hypnozoites in the liver of MMV048-treated mice and large schizonts in DMSO-treated controls (Fig. 1*B*). Quantification of the number of schizonts and hypnozoites showed that MMV048-treated mice from groups 2, 3, and 4 contained a significantly lower number of schizonts and similar number of hypnozoites when compared to respective untreated control mice (Fig. 1*C*). Notably, livers from intravenous-infected mice analyzed at day 8 showed a larger number of liver-stage forms when compared to mice from MB infections. Livers collected after 21 days post-IV showed no schizonts in both MMV048 and DMSO-treated mice, and only one hypnozoite was detected in MMV048-treated mice. These results indicate that hypnozoites failed reactivation after 8 days and a second generation of schizonts did not form. Quantification by RT-qPCR analysis of 18S rRNA in liver tissue showed a significantly reduced parasite load in MMV048-treated mice when compared to DMSO-treated controls in both mosquito-bite infected and IV analyzed 8 dpi (Fig. 1*D*), supporting the results observed by IFA. In spite of the absence of liver stages in IFA at day 21, parasite load was found similar to levels of hypnozoites-enriched livers from group 3 with no differences in MMV48 and DMSO-treated animals.

As expected for radical cure treatment, no liver forms were quantified in the liver of TQ-treated animals, although RT-qPCR data indicated presence of parasite 18S rRNA in this condition. Plasma from *P. vivax*-infected FRG huHEP mice from all experimental groups was used as a source of circulating EVs which were further isolated by SEC (43). Molecular characterization of SEC fractions by flow cytometry bead–based assay showed an enrichment of CD9^+^ and CD5L^+^ vesicles between fraction 7, 8, and 9, a profile that is in agreement with small vesicles or exosomes elution profiles reported by qEV columns manufacturer (supplemental Fig. S1, *A*–*E*). Importantly, enriched vesicles were clearly separated from the bulk of plasma soluble proteins as estimated from the protein concentration. Additional analysis of CD9^+^ CD5L^+^ enriched SEC fractions showed the presence of CD63 and CD81 (supplemental Fig. S2). Particles size distribution and concentration of the highest CD5L/CD9/CD63 SEC fractions quantified by NTA showed that enriched particles had a size between 55 to 100 nm (mode) and 75 to 120 (mean) and a concentration in the range of 5 × 10^8^ and 1.2 × 10^9^ particles/ml (supplemental Fig. S3). Particle size corresponds to that one of small vesicles which includes exosomes and plasma membrane-derived smalls EVs (19). Complementary analysis by cryo-transmission electron microscopy of EVs enriched SEC fractions from experimental group 2 mice (MB-D8) showed the presence of single membrane electron dense nanovesicles of homogenous size [mean size: DMSO-treated 152.17 nm and MMV048-treated 144.5 nm] (supplemental Fig. S4).

**Fig. 2:**
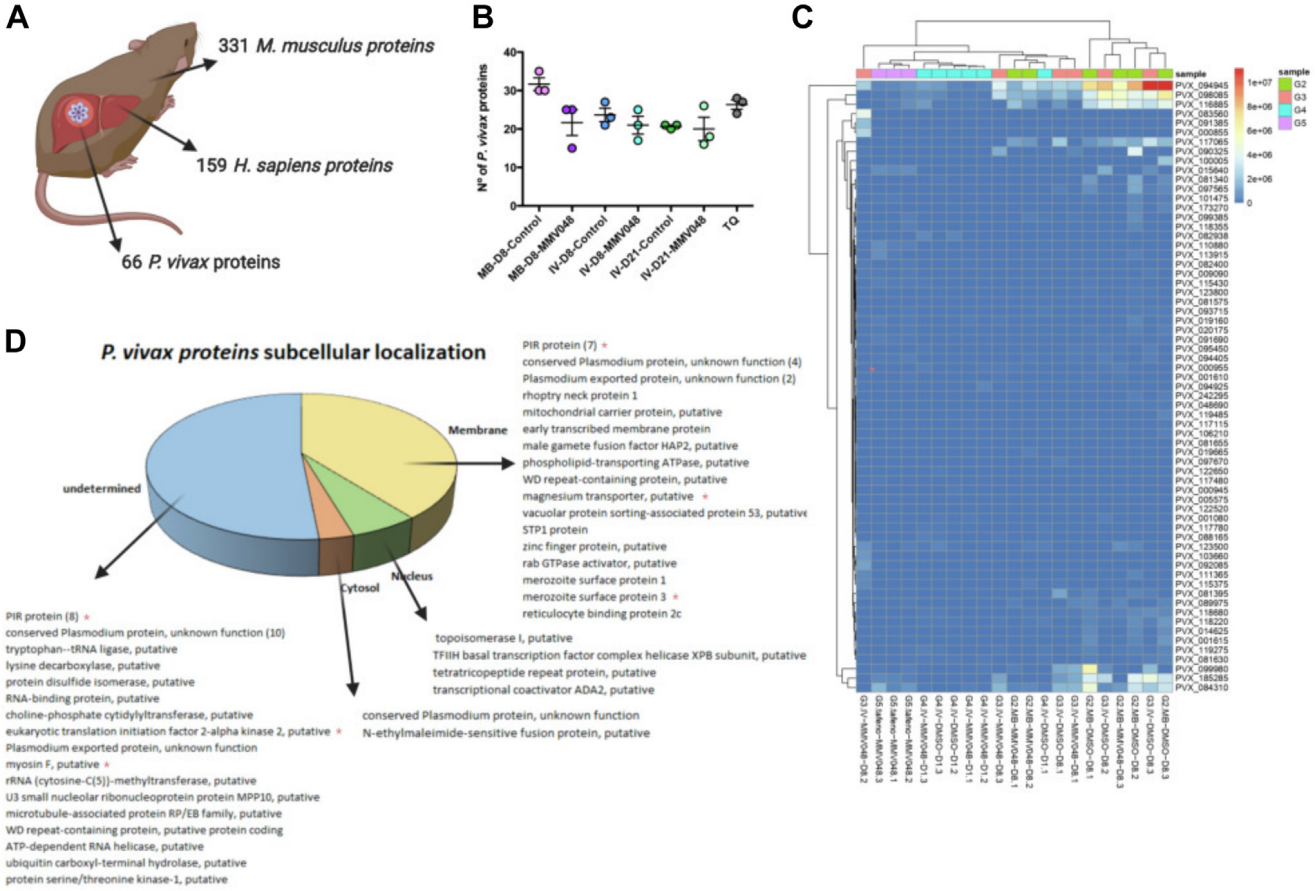
**Identification of *P. vivax* hypnozoites biomarkers associated with plasma-derived EVs in the FRGhuHEPmice *in vivo* model.***A*, total proteins identified from *H. sapiens*, *M. musculus*, and *P. vivax* in plasma-derived EVs from all experimental groups. *B*, number of *P. vivax* proteins identified in plasma-derived EVs from each experimental group. Data represent mean and standard error of *P. vivax* proteins identified in mice from each group. *C*, heat map of estimated protein abundances (AEpS) showing hierarchical clustering of parasite EV proteins across the infected FRG huHEP mice. *D*, distribution *P. vivax* proteins in different subcellular compartments as predicted by GO enrichment terms and Uniprot cell compartment assignments. Membranes, cytosol, nucleus, and undetermined compartments are represented. *Red asterisk* (∗) refers to proteins with no orthologues in *P. falciparum*. *A* has been created with BioRender.com. EVs, extracellular vesicles.

Proteomic characterization of plasma-derived EVs from all groups of *P. vivax*-infected FRG huHEP mice showed that EVs-enriched SEC fractions contained proteins from the three species: 159 human, 331 mouse, and 66 *P. vivax* proteins (Fig. 2*A*) (supplemental Table S1). Human proteins include 18 proteins from the top 100 EVs most abundant proteins as reported by Vesiclepedia (44). From the whole human proteome obtained, 20, 63, and 23 proteins were previously reported in proteomes from human hepatocytes (45, 46, 47), respectively, and 77 in *P. vivax* infected FRG huHEP mice (37) (supplemental Table S2). EVs markers from mouse origin included CD9, CD5L, syntenin-1, integrin a and b, and Na/K ATPase and from human origin heat shock cognate 71, annexin A2, 14-3-3 protein epsilon, peroxiredoxin-1, Rap-1b, and Rab-10, among others.

The number of *P. vivax* proteins identified were similarly distributed in all experimental groups, with a tendency of smaller number of proteins in EVs from MMV048-treated mice of MB-D8 (group 2) and IV-D8 (group 3) when compared to DMSO-treated controls, although not statistically significant different (Fig. 2*B*). Hierarchical cluster heatmap shows that EVs proteins from infected mice were relatively well segregated according to the treatment (Fig. 2*C*). Two clusters of *P. vivax* proteins showed correlation in mice with active schizont replication (DMSO-treated mice analyzed at day 8) independent of the infection mode. The first cluster include transcriptional coactivator ADA2, putative topoisomerase I, and a hypothetical protein with unknown function (PVX116885), while the second included merozoite surface protein 1, one PIR protein (PVX_185285), and a putative tetratricopeptide repeat protein (PVX_084310) (Fig. 2*C*). Subcellular localization prediction indicates that 39% of the total proteins identified were associated to membranes, 3% to cytosol, and 6% to nucleus (Fig. 2*D*). The remaining 52% proteins were not assigned to a specific cell compartment in our analysis. These proteins include eight members of the VIR family, ten conserved *Plasmodium* proteins of unknown function, and several proteins with a large variety of predicted functions. Membrane proteins include seven VIR family members, an early transcribed protein (ETRAMP), vacuolar sorting-associated protein 53, Rab GTPase activator, reticulocyte binding protein 2c, and merozoite surface protein 1 and 3, among others. Cytosolic proteins include a conserved *Plasmodium* protein of unknown function and N-ethylmaleimide-sensitive fusion protein involved in protein transport. Nuclear proteins include proteins associated to transcription like TFIIH basal transcription factor complex helicase XPB subunit and the abovementioned topoisomerase I, tetratricopeptide repeat protein, and transcriptional coactivator ADA2 (Fig. 2*D* and supplemental Table S1). Interestingly, 20 of the *P. vivax* proteins identified were found to be specific to the hypnozoite-forming species (Fig. 2*D*).

Screening of hypnozoites-specific proteins associated to EVs was done by first comparing the PR of *P. vivax* proteins across the different experimental groups (supplemental Table S3). We excluded proteins identified in the group of mice treated with tafenoquine and then compared MMV048-treated infected and DMSO-treated control–infected mice in each experimental group. We performed the same analysis in the AEpS data of *P. vivax* proteins shown in supplemental Table S1. This screening showed the presence of 13 potential candidate proteins. These were detected in MMV048-treated mice which plasma sample was collected after 8 (MB and IV infection) and 21 dpi and absent in its respective intragroup DMSO-treated controls (supplemental Table S4). As these set of proteins were identified with one unique peptide, we performed manual inspection to evaluate the compatibility of the detected experimental spectra with its corresponding theoretical predicted spectra using MS2PIP software (48). This analysis showed spectra compatibility for only four proteins (supplemental Table S4 and supplemental Fig. S5). These proteins correspond to a conserved Plasmodium protein of unknown function (PVP01_1103700), a phospholipid-transporting ATPase, putative (PVP01_1441600), a PIR protein (PVP01_0006080), and filamin domain-containing protein, putative (PVP01_0915600). It should be noticed, however, that from these four potential candidates, only PVP01_0915600 was exclusive to MMV048 treatment in the intergroup comparison.

Interestingly, one protein (PVP01_0814300) which corresponds to HAP2, a well-conserved *Plasmodium* protein previously found to be involved in membrane fusion during fertilization and important for parasite transmission (49), was found in three biological replicates of the treated mice from group 2 (MB-MMV048-8D) and in one mouse of the treated group 4 (IV-MMV048-21D); however, we found no compatibility in the experimental/theoretical peptide spectra analysis.

**Fig. 3:**
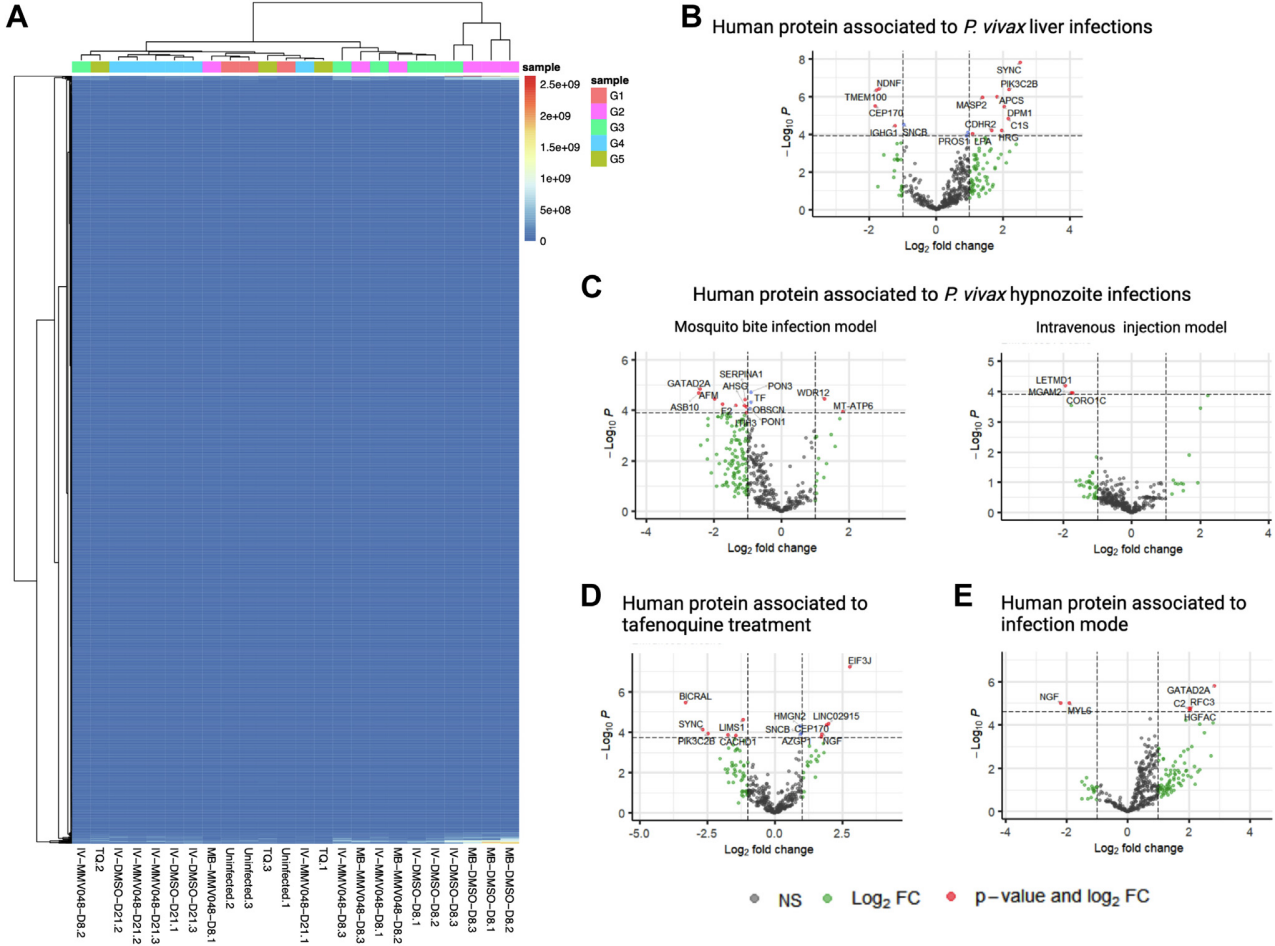
**Human proteome of plasma-derived EVs from *P. vivax*-infected FRGhuHEPmice.***A*, heat map of estimated protein abundances (AEpS) showing hierarchical clustering of human EV protein cargo across the experimental groups of FRG huHEP mice. *B*–*E*, enhanced volcano plots showing comparisons of human EV protein cargo between various groups as indicated in statistical analysis of materials and methods. *B*, human EV proteins associated with *P. vivax* liver infection {upregulation [positive fold change (FC), downregulation (negative FC) upon infection]}. *C*, human EV proteins associated with *P. vivax* hypnozoites infection in the mosquito bite (*left*) and intravenous (*right*) infection mode [upregulation (positive FC), downregulation (negative FC) in MMV048-treated mice]. *D*, human EV proteins associated with tafenoquine treatment of *P. vivax*-infected mice. Upregulation (positive FC), downregulation (negative FC) in DMSO-treated mice. *E*, human EV proteins associated with infection mode. Upregulation (positive FC), downregulation (negative FC) in mosquito bite–infected mice. The plot was constructed using −log10 (*p* value) against the estimate (Log2 FC). *Dotted horizontal line* represents *p* value = 0.05. Proteins with an estimate ±2-fold and adjusted *p* value <0.05 are named and shown as *red dots*. In addition, upregulated and downregulated proteins with adjusted *p* value greater than 0.05 are represented by *green dots*. Supporting information of this figure can be found in supplemental Tables S6–S8. EVs, extracellular vesicles.

To determine if human proteins secreted in circulating EVs from *P. vivax* infected FRG huHEP mice can reveal particular biological aspects of liver infection, we performed a statistical analysis of human proteomic data and compared AEpS from different experimental groups. First, we analyzed the per-sample human protein abundance distributions. Supplemental Fig. S6 shows that all groups lie within a similar range. Hierarchical cluster heatmaps of human EV proteins identified in all groups show good segregation of samples according to the different treatments (Fig. 3*A*). However, we did not observe correlation in the great majority of proteins with the exception of two clusters which correlate with active schizont replication (DMSO-treated mice) at 8 dpi. Next, to identify human proteins associated with infection, we interrogated the totality of human proteins detected in uninfected and DMSO-treated mice from groups 2, 3, and 4 (supplemental Table S5). In total, 15 human proteins were found to be significantly (adjusted *p* value < 0.05) altered according to infection status, six showing downregulation and nine showing upregulation upon infection (Fig. 3*B*). Upregulated proteins are involved in negative regulation of endopeptidase activity, fibrinolysis, proteolysis, complement activation, and platelet degranulation as inferred from gene ontology analysis (supplemental Table S6), likely reflecting early inflammatory processes in response to infection. To identify a possible human biomarker of hypnozoite infection, we contrasted the protein abundance of proteins identified in EVs from MMV048 *versus* DMSO-treated animals within experimental groups 2 and 3. This analysis showed that EVs from MB infections showed larger changes in differentially expressed human proteins upon treatment when compared to IVs (Fig. 3*C*). These changes included upregulation of 12 (MB) and three (IV) proteins in DMSO-treated mice while only two proteins were upregulated in MMV048-treated mice (MB) (Fig. 3*C*). Gene ontology enrichment analysis showed human EVs cargo from mice with active schizont replication in this comparison correspond to biological processes related to acute inflammatory immune response (supplemental Table S7). This indicates that liver hypnozoite infections do not elicit systemic EVs hallmarks of inflammation when compared to schizont infections. In spite of upregulation of two proteins in EVs from MMV048-treated mice, a postanalysis showed that in an intergroup comparison, these candidates were not uniquely identified in MMV048-treated mice, therefore precluding its designation as human biomarkers of hypnozoite infection. Further, we compared human proteins found in plasma-derived EVs from TQ-treated *versus* DMSO-treated mice from groups 2, 3, and 4. This comparison showed five proteins upregulated upon TQ treatment (Fig. 3*D* and supplemental Table S8). Interestingly, one upregulated protein corresponds to phosphatidylinositol-4-phosphate 3-kinase catalytic subunit type 2 beta, an enzyme involved in the phosphatidylinositol phosphate metabolism in several cell compartments from a wide variety of tissues, notably the liver. Additionally, we compared human proteins from mice infected by artificial intravenous injection with mice infected by MB. Among the few proteins that were found statistically different, GATA zinc finger domain containing 2A, a plasma protein with transcription factor activity, was found to be 2.8 times upregulated in natural infection mode by MB (Fig. 3*E* and supplemental Table S8).

## Discussion

Here, we explored the proteome of EVs derived from plasma of MMV048-treated FRG huHEP mice infected with *P. vivax* sporozoites in which there was an enrichment of *P. vivax* hypnozoites forms.

MMV048 has been proved to have potent activity against asexual, transmission, and liver stages of *Plasmodium* spp. in preclinical studies (50). The target of this drug is the PI4K, a protein involved in membrane recruitment and dynamics during asexual replication (51). Interestingly, when applied in radical cure mode, this drug has been proved ineffective against liver hypnozoites in *P. cynomolgi*, both *in vitro* and *in vivo* (50), likely due to the low expression of the PI4K drug target in this parasite stage, as has been previously suggested for *P. vivax* hypnozoites (13). Thus, MMV048 should eliminate all replicating *P. vivax* liver stages while leaving hypnozoites unaffected, a result that was observed in infected FRG huHEP mice (Fig. 1, *B*–*D*), thus validating this approach for searching hypnozoite biomarkers.

Overall proteomic analysis of plasma-derived EVs in the FRG huHEP mice model suggested the presence of 66 potential parasite proteins. These included proteins involved in lipids and ions transport, such as a putative mitochondrial carrier protein and phospholipid-transporting ATPase as well as a putative magnesium transporter. In addition, we detected proteins participating in membrane trafficking like the vacuolar sorting–associated proteins p53 putative and Rab GTPase activator (Fig. 2*D*). Metabolic enzymes and DNA remodeling and RNA binding proteins were also present. This agrees with a functional association with EVs as these nanovesicles are characterized by the presence of multiple membrane, cytosolic, and nuclear proteins. Of interest, a large component of the parasite proteome was represented by several members of the variant *vir* gene superfamily. The association of VIR proteins with EVs could be due to its sorting in host-derived EVs. Of note, several proteins identified in plasma-derived EVs from infected FRG huHEP mice are also found in merozoites stages (MSP1, MSP3, RBP2c, Rhoptry neck protein) (Fig. 2*D*). Such association with EVs could indicate that hepatocytes infected with mature exo-erythrocytic schizonts secrete EVs that reach circulation. Importantly, the biogenesis pathway of *P. vivax* liver stages-derived EVs is presently unknown and therefore validation of the proteins identified in this study cannot be contrasted with specific liver stage EV markers, an important limitation in the field.

To identify hypnozoite-specific proteins associated with EVs, we mined the proteome data from MMV048-treated mice and performed an analysis involving intragroup and intergroup comparisons across all experimental conditions followed by a peptide compatibility analysis with predicted spectra to warrant robust identification. Only one protein fulfilled this stringent top-down selection, a well conserved filamin domain–containing protein, putative (PVP01_0915600). Filamin proteins in mammals binds to actin and crosslink cytoskeleton to glycoproteins in the membrane, regulating cell shape and migration (52). Whether this putative filamin domain containing protein perform a similar role in *Plasmodium* parasites is unknown. Importantly, filamin family members have been found in EVs from a vast range of cells types in mammals (44). Of note, previous reports on hypnozoites transcriptional analysis failed to identify transcriptional activity of the gene coding for this protein (13, 53). Further studies of this unique potential hypnozoite biomarker are warranted.

Although the predicted spectrum is not compatible with the identified peptide sequence for HAP2, a potential peptide identification of this protein appeared in all three biological replicates of MMV048-treated mice infected by MBs as well as in one mouse infected by intravenous injection and analyzed after 21 dpi. Interestingly, HAP2 is a well conserved protein that is express in male gametes of malaria parasites that has a structural similarity to the class II viral fusion proteins involved in fusogenic membrane process in a wide range of organisms including plants, nonpathogenic and pathogenic protists (49). Hypnozoite expression of HAP2 in *P. vivax* is supported by a previous transcriptional analysis that has shown that this gene is expressed in hypnozoite-enriched–infected hepatocyte cultures as compared to a schizont/hypnozoite mix culture (13). Moreover, in the most recent transcriptional analysis of *P. vivax* liver stages, it was found that a subpopulation of nonreplicating hypnozoites is sexually committed which provides additional biological support to the potential detection of HAP2 in EVs derived from hypnozoites. The presence of a transmembrane domain and its molecular function in membrane fusion events indicates that HAP2 association with EVs is plausible and could play biological functions in the parasite EVs biogenesis process. The fact that HAP2 is also expressed in other *Plasmodium* spp., could compromise its potential value as a biomarker specific for *P. vivax* hypnozoites. However, we found a C-terminal peptide exclusively present in hypnozoite-forming *Plasmodium* species, indicating that it might be possible to develop species-specific reagents to detect HAP2 only from relapsing malaria species. More studies will be necessary to confirm the presence of HAP2 in EVs-derived from hypnozoites in this *in vivo* model.

The potential of EVs molecular contents as biomarkers of disease is attributed to the fact that they are a fingerprint of the cell of origin (44). In this sense, the assessment of statistical differences between the human components of plasma-derived EVs in the FRG huHEP mice throughout the different experimental conditions used in this study aimed to gain additional knowledge of the physiological status of human hepatocytes during this infection. Our results reflect that infected hepatocytes respond to *P. vivax* infection secreting EVs with signatures of inflammation (Fig. 3*B*) being hypnozoite infections immunologically silent when compared to replicating schizonts (Fig. 3*C*). These data are therefore in agreement with previous results demonstrating that liver infections induced an inflammatory response in the host (54). Noteworthy, we found upregulation of a member of the PI3K family (PIK3C2B) in EVs from tafenoquine-treated mice when compared to infected and DMSO-treated mice (Fig. 3*D*). This could imply that tafenoquine may induce increased expression and secretion in EVs of PIK3C2B provoking alterations in hepatocytes cell-signaling pathways involved in proliferation and cell survival, an intracellular protein trafficking during tafenoquine metabolism in the liver.

In summary, our results show that LC-MS/MS-based proteomics of EVs generated in this *in vivo* model of *P. vivax* upon treatment with the schizonticidal experimental drug MMV048 identified parasite proteins secreted in EVs from infected human hepatocytes. Moreover, human proteins identified in plasma-derived EVs from human liver chimeric FRG huHEP mice represent the content of a subpopulation of EVs coming exclusively from human hepatocytes without the confounding of other human cells. Such information can be useful for future studies of EVs in the context of liver diseases. Last, this study set the stage to further discover specific biomarkers of asymptomatic *P. vivax* liver infections associated with EVs and should advance the development of diagnostic tools for the identification of asymptomatic hypnozoite carriers in human populations.

## Data Availability

All data described in the manuscript are contained within the manuscript and the supplemental data.

EV isolation and characterization: We have submitted all relevant data of our experiments to the EV-TRACK knowledgebase (EV-TRACK ID: EV200176) (55).

Mass spectrometry proteomic data: We have deposited data to the ProteomeXchange Consortium *via* PRIDE partner repository (56) with the dataset identifier PXD035719.

## Supplemental data

This article contains supplemental data.

## Conflict of interest

V. C., S. A. M., and E. L. F. are employed by and/or is a shareholder of Novartis Pharma AG. Other authors declare that the research was conducted in the absence of any commercial or financial relationships that could be construed as a potential conflict of interest.
