# Supplementary material for: Mass Spectrometry Identification of Biomarkers in Extracellular Vesicles From Plasmodium vivax Liver Hypnozoite Infections
Source: Mol Cell Proteomics. 2022 Aug 24;21(10):100406. doi: 10.1016/j.mcpro.2022.100406 (PMC9520272; doi:10.1016/j.mcpro.2022.100406)
Supplement: Supplemental Figures S1–S6 [file mmc1.docx]

**Supplemental Information**

**Mass spectrometry identification of biomarkers in extracellular vesicles from *Plasmodium vivax* liver hypnozoite infections**

Authors: Melisa Gualdrón-López^1,2+^, Miriam Díaz-Varela^1,2++^, Gigliola Zanghi^3^, Iris Aparici-Herraiz^1,2^, Ryan W. J. Steel^3+++^, Carola Schäfer^3++++^, Pol Cuscó^1^, Vorada Chuenchob^3+++++^, Niwat Kangwangransan^4^, Zachary P. Billman^5^, Tayla M. Olsen^5^, Juan R. González^1^, Wanlapa Roobsoong^6^, Jetsumon Sattabongkot^6^, Sean C. Murphy^5^, Sebastian A. Mikolajczak^3+++++^, Eva Borràs^7,8^, Eduard Sabidó^7,8^, Carmen Fernandez-Becerra^1,2^, Erika L. Flannery^3+++++^, Stefan H. I. Kappe^3,9^ and Hernando A del Portillo^1, 2,10*^

**Supplemental information file content**

1. Supplementary Excel file 1. Proteomic data of plasma-derived EVs from P. vivax infected FRG huHep mice. (Submitted as a separate Excel file).

2. Supplementary Excel file 2. Statistical analysis of human proteins identified in plasma-derived EVs from P. vivax infected FRG huHEP. (Submitted as a separate Excel file).

3. Supplementary figure 1

4. Supplementary figure 2

5. Supplementary figure 3

6. Supplementary figure 4

7. Supplementary figure 5

8. Supplementary figure 6

**Supplementary figures**


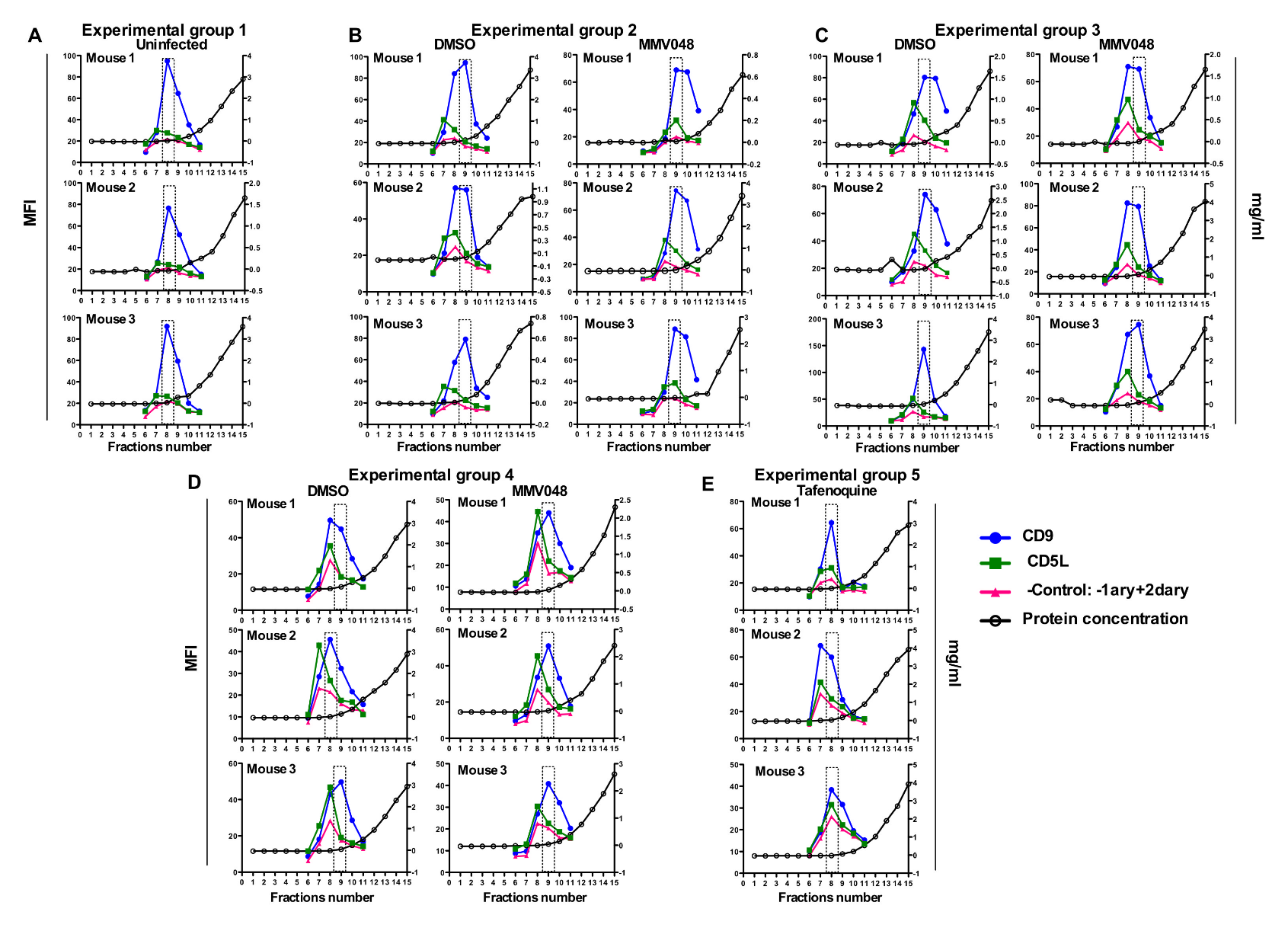


**Fig S1. Molecular characterization of plasma-derived EVs from *P. vivax* infected FRG huHep mice treated with MMV048 by flow cytometry.** Plots shows individual *P. vivax* infected FRG huHep mice Size Exclusion Chromatography (SEC) profiles. SEC fractions were analyzed for the presence of CD9 and CD5L EVs marker in a flow cytometry bead-based assay. Negative control refers to fractions F6-F11-conjugated beads incubated with Alexa 488- secondary (2ary) antibody. *A* Experimental group 1: Uninfected mice; *B* Experimental group 2: Mosquito-bite infected mice at 8 dpi; *C* Experimental group 3: Intravenous infected mice at 8 dpi; *D* Experimental group 4: Intravenous infected mice at 21 dpi and *E* Intravenous infected mice treated with Tafenoquine at 21 dpi.


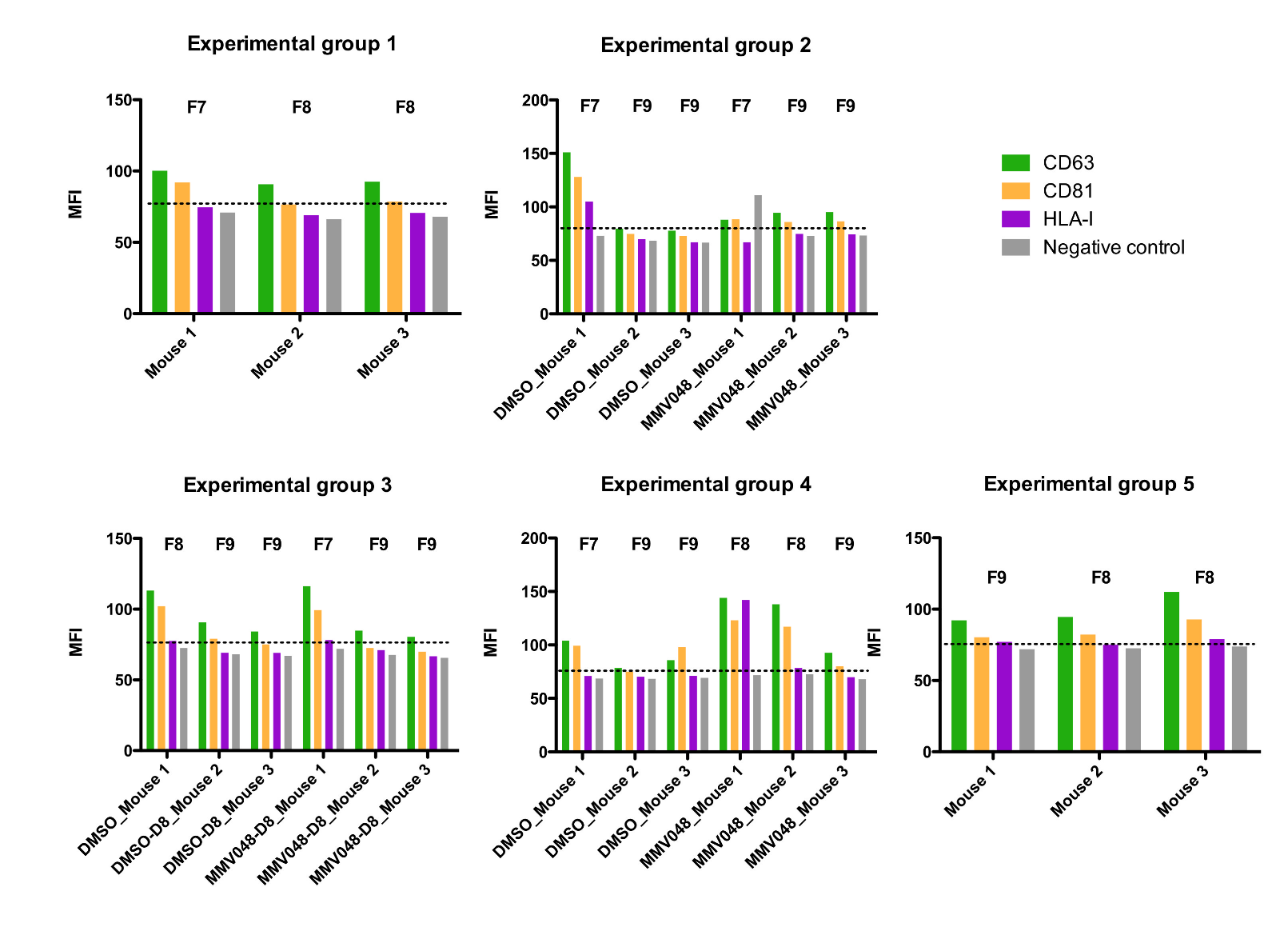
**Fig S2. Molecular analysis of additional EVs markers in CD9^+^CD5L^+^ single pick SEC fractions from plasma-derived EVs from *P. vivax* infected FRG huHEP mice**. CD9^+^CD5L^+^ single pick SEC fractions from individual mice were further tested for CD63, CD81 and HLA-I by BBA. Negative control refers to CD9^+^CD5L^+^ single pick SEC fraction conjugated-beads incubated with Alexa 488 (secondary) 2ary antibody in the absence of primary (1ary) antibodies. Cut-off values were determined as the mean plus two-fold standard deviation from all samples.


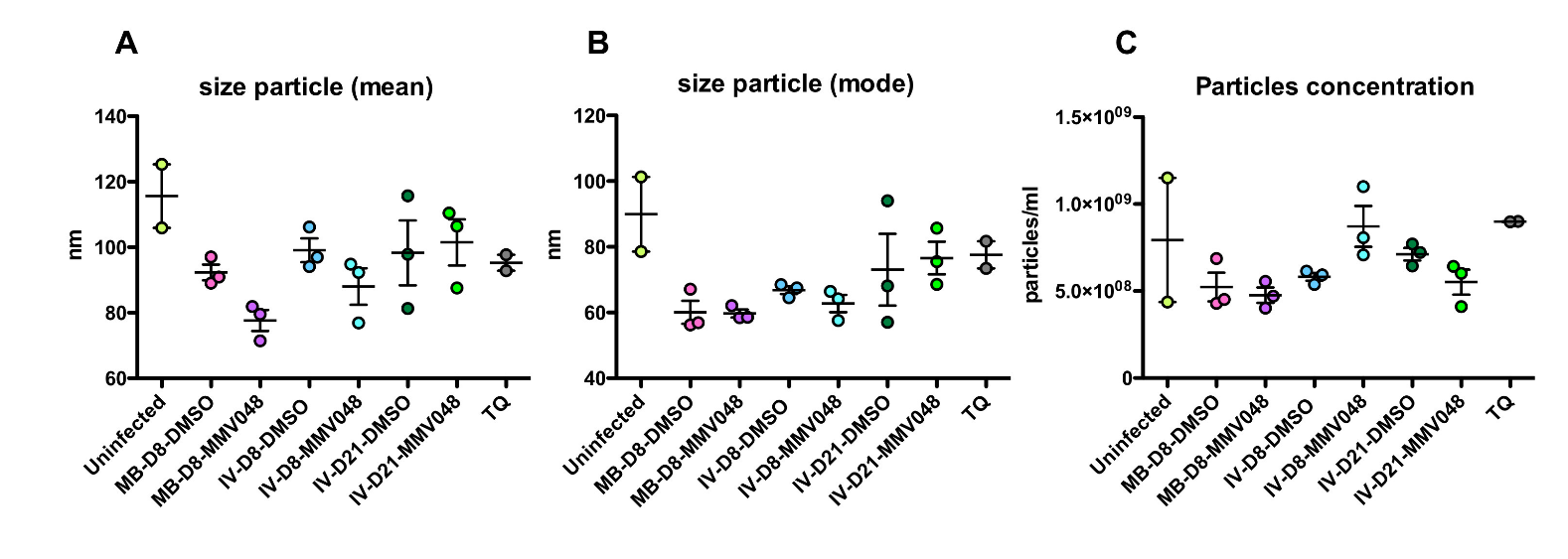
**Fig S3. Nanoparticle track analysis of plasma-derived EVs from *P. vivax* infected FRG huHEP mice.** Plots show size mode (A), mean (B) and concentration (C) of particles from CD5L^+^/CD9^+^ pick SEC fractions from all experimental groups. Data represent mean and standard error of individual measurements performed in each mouse. Statistically significant differences between groups was tested in a paired t-test. No differences were found.


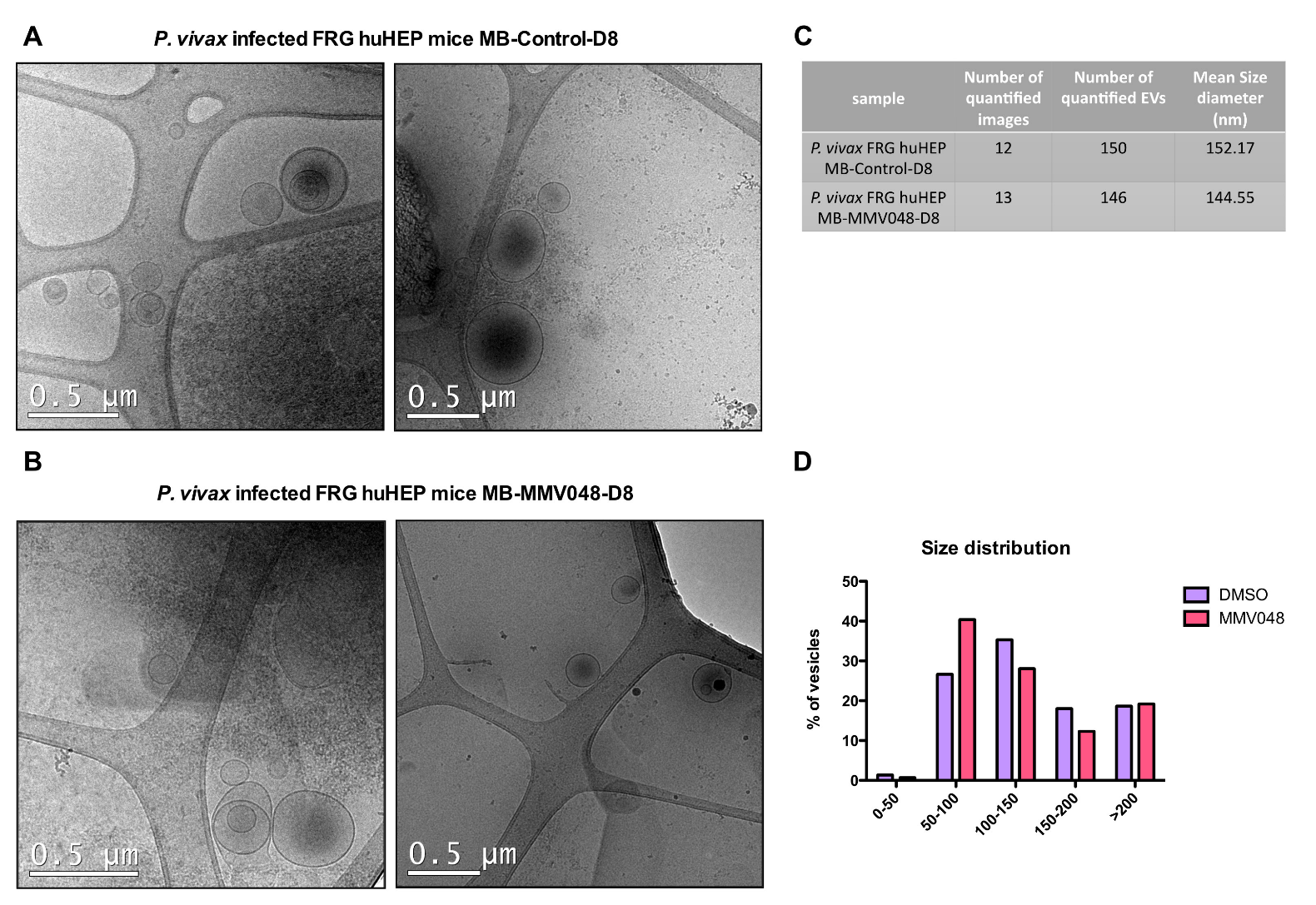


**Fig. S4. Cryo-TEM analysis of plasma-derived EVs from *P. vivax*-infected FRG huHEP mice.** Images showing isolated EVs from plasma of *P. vivax* infected FRG huHEP mice by mosquito-bite and treated with DMSO (A) or MMV048 (B). EVs diameter were quantified using ImageJ (NIH) where pixels were calibrated to nanometers (C). Mean diameter obtained after analysis of 12-13 pictures/condition is shown. (D) EVs size distribution.


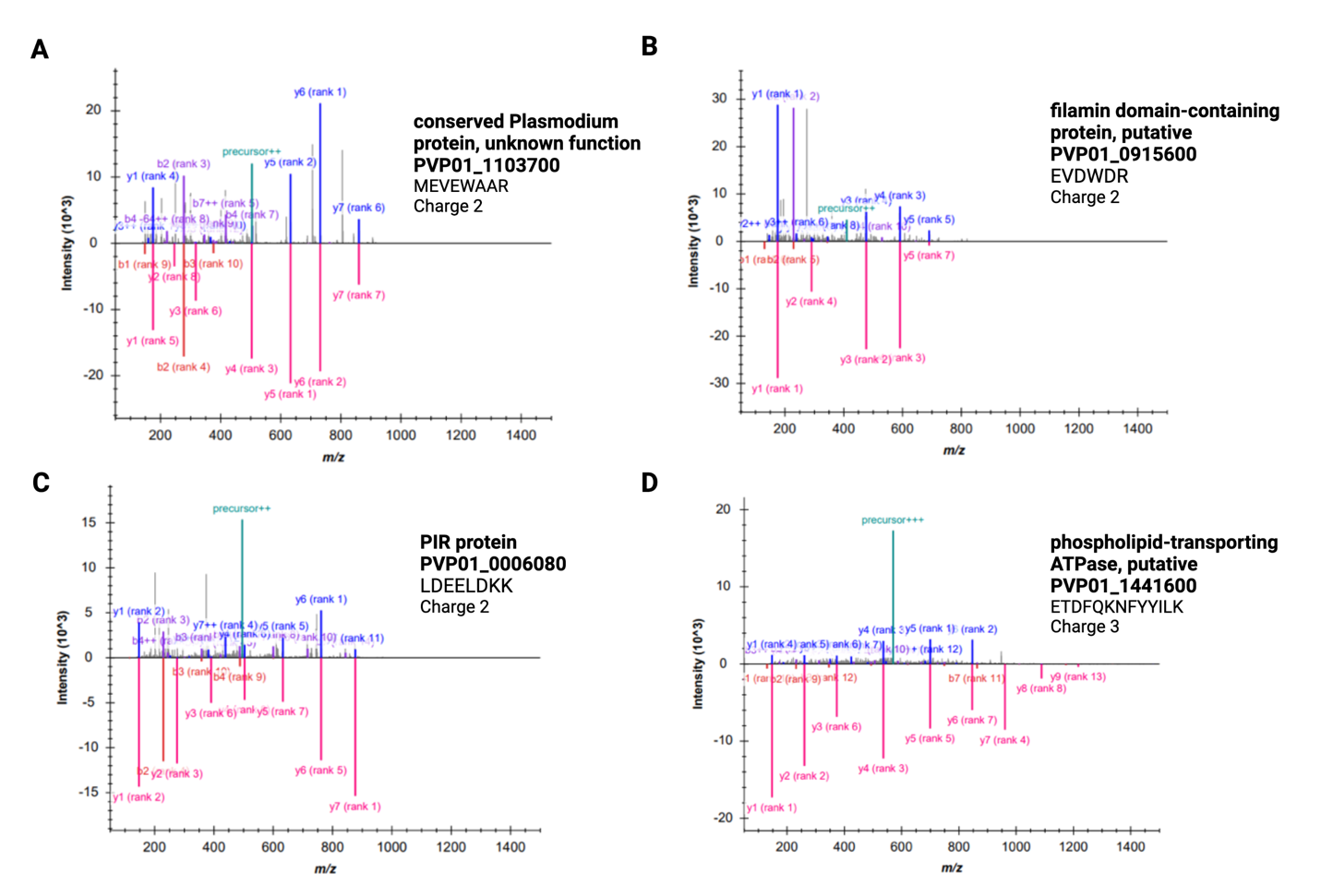


**Fig S5. Experimental versus predicted spectra compatibility analysis of peptides from proteins identified in EVs from MMV048 treated *P. vivax* infected FRG huHEP mice.** In each panel, the top spectra (blue) is the experimental one and the bottom (red) the predicted by MS2PIP


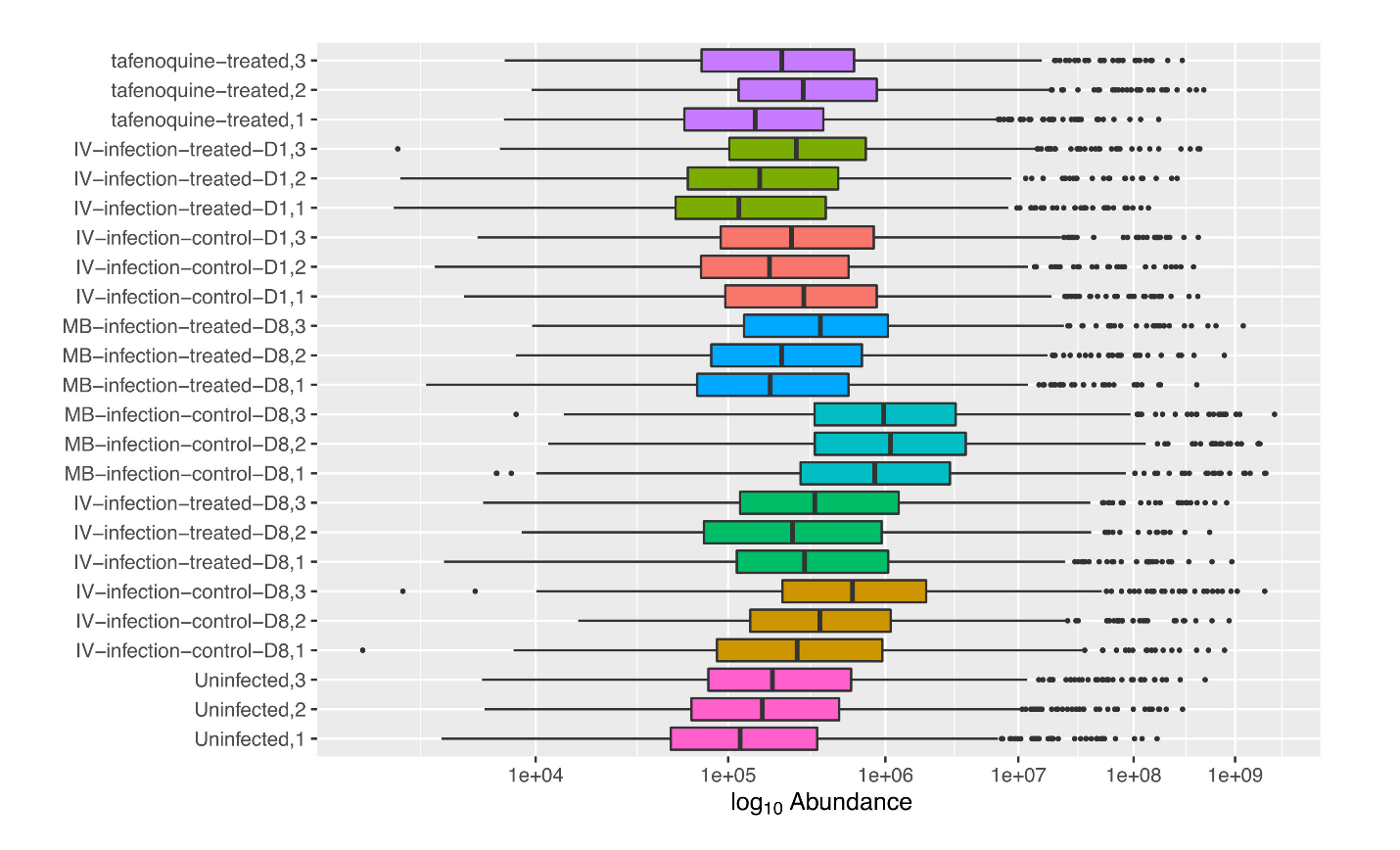


**Fig S6. Abundance distribution of human proteins in plasma-derived EVs from *P. vivax* infected FRG huHEP mice**. Plots shows that per sample log10 protein abundance distribution usually lie within a similar range.
